# Complete genome sequence of *Catenulispora acidiphila* type strain (ID 139908^T^)

**DOI:** 10.4056/sigs.17259

**Published:** 2009-09-24

**Authors:** Alex Copeland, Alla Lapidus, Tijana Glavina Del Rio, Matt Nolan, Susan Lucas, Feng Chen, Hope Tice, Jan-Fang Cheng, David Bruce, Lynne Goodwin, Sam Pitluck, Natalia Mikhailova, Amrita Pati, Natalia Ivanova, Konstantinos Mavromatis, Amy Chen, Krishna Palaniappan, Patrick Chain, Miriam Land, Loren Hauser, Yun-Juan Chang, Cynthia D. Jeffries, Olga Chertkov, Thomas Brettin, John C. Detter, Cliff Han, Zahid Ali, Brian J. Tindall, Markus Göker, James Bristow, Jonathan A. Eisen, Victor Markowitz, Philip Hugenholtz, Nikos C. Kyrpides, Hans-Peter Klenk

**Affiliations:** 1 DOE Joint Genome Institute, Walnut Creek, California, USA; 2 Los Alamos National Laboratory, Bioscience Division, Los Alamos, New Mexico, USA; 3 Biological Data Management and Technology Center, Lawrence Berkeley National Laboratory, Berkeley, California, USA; 4 Lawrence Livermore National Laboratory, Livermore, California, USA; 5 Oak Ridge National Laboratory, Oak Ridge, Tennessee, USA; 6 DSMZ - German Collection of Microorganisms and Cell Cultures GmbH, Braunschweig, Germany; 7 University of California Davis Genome Center, Davis, California, USA

**Keywords:** acidophilic, free-living, vegetative and aerial mycelia, filamentous actinomycete, non-pathogenic, aerobic, *Catenulisporineae*

## Abstract

*Catenulispora acidiphila* Busti *et al*. 2006 is the type species of the genus *Catenulispora*, and is of interest because of the rather isolated phylogenetic location it occupies within the scarcely explored suborder *Catenulisporineae* of the order *Actinomycetales*. *C. acidiphilia* is known for its acidophilic, aerobic lifestyle, but can also grow scantly under anaerobic conditions. Under regular conditions, *C. acidiphilia* grows in long filaments of relatively short aerial hyphae with marked septation. It is a free living, non motile, Gram-positive bacterium isolated from a forest soil sample taken from a wooded area in Gerenzano, Italy. Here we describe the features of this organism, together with the complete genome sequence and annotation. This is the first complete genome sequence of the actinobacterial family *Catenulisporaceae*, and the 10,467,782 bp long single replicon genome with its 9056 protein-coding and 69 RNA genes is a part of the *** G****enomic* *** E****ncyclopedia of* *** B****acteria and* *** A****rchaea * project.

## Introduction

**Figure 1:**
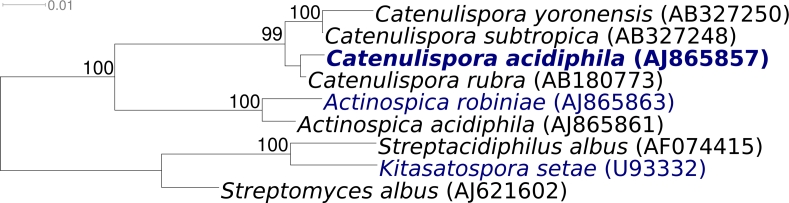
Phylogenetic tree of *C. acidiphila* ID 139908^T^ and all type strains of the genus *Catenulispora*, inferred from 1,421 aligned characters [3,4] of the 16S rRNA sequence under the maximum likelihood criterion [5]. The tree was rooted with the type strains of the genera within the *Streptomycetaceae* (*Streptomycineae*, *Actinomycetales*). Also included are the type strains from the sister family of *Catenulisporaceae*, *Actinospicaceae*. The branches are scaled in terms of the expected number of substitutions per site. Numbers above branches are support values from 1,000 bootstrap replicates if larger than 60%. Strains with a genome sequencing project registered in GOLD [6] are printed in blue; published genomes in bold.

*Catenulispora acidiphila* strain ID 139908^T^ (= DSM 44928 = NRRL B-24433 = JCM 14897) is the type species of the genus *Catenulispora* which is the type genus of family *Catenulisporaceae,* as well as of the suborder *Catenulisporineae* [1]. The *Catenulisporacineae* is a rather small (six genera in two families) and young taxon [2], for which no completed genome sequence has been reported to date (Figure 1). The four *Catenulispora* type strains were isolated from paddy field or forest soil, prefer slightly acidic habitats, and form vegetative and aerial mycelia [1,7,8]. Here we present a summary classification and a set of features for *C. acidiphila* ID 139908^T^ (Table 1), together with the description of the complete genomic sequencing and annotation.

**Table 1:** Classification and general features of *C. acidiphila* ID 139908^T^ according to the MIGS recommendations [9]

**MIGS ID**	**Property**	**Term**	**Evidence code**
	Current classification	Domain *Bacteria*	TAS [10]
		Phylum *Actinobacteria*	TAS [11]
		Class *Actinobacteria*	TAS [12]
		Order *Actinomycetales*	TAS [12]
		Suborder *Catenulisporineae*	TAS [2]
		Family *Catenulisporaceae*	TAS [1]
		Genus *Catenulispora*	TAS [1]
		Species *Catenulispora acidiphila*	TAS [1]
		Type strain ID 139908	TAS [1]
	Gram stain	positive	TAS [1]
	Cell shape	non-fragmentary vegetative mycelium	TAS [1]
	Motility	nonmotile	TAS [1]
	Sporulation	produces arthrospores when induced	TAS [1]
	Temperature range	mesophilic, 11-37°C	TAS [1]
	Optimum temperature	22-28°C	TAS [1]
	Salinity	3% NaCl	TAS [1]
MIGS-22	Oxygen requirement	essentially aerobic; capable of reduced and non-pigmented growth under microaerophilic and anaerobic conditions	TAS [1]
	Carbon source	glucose, arabinose, xylose, mannitol, fructose, glycerol	TAS [1]
	Energy source	starch	NAS
MIGS-6	Habitat	soil	TAS [1]
MIGS-15	Biotic relationship	free living	NAS
MIGS-14	Pathogenicity	none	NAS
	Biosafety level	1	TAS [13]
	Isolation	forest soil from wooden area	TAS [2]
MIGS-4	Geographic location	Gerenzano, Italy	TAS [2]
MIGS-5	Sample collection time	before 2006	TAS [1]
MIGS-4.1 MIGS-4.2	Latitude, Longitude	45.640, 9.002	NAS
MIGS-4.3	Depth	not reported	
MIGS-4.4	Altitude	not reported	

Evidence codes - IDA: Inferred from Direct Assay (first time in publication); TAS: Traceable Author Statement (i.e., a direct report exists in the literature); NAS: Non-traceable Author Statement (i.e., not directly observed for the living, isolated sample, but based on a generally accepted property for the species, or anecdotal evidence). These evidence codes are from the Gene Ontology project [14]. If the evidence code is IDA, then the property was observed for a living isolate by one of the authors or an expert mentioned in the acknowledgements.

## Classification and features

The strains most probably belonging to the species *C. acidiphila* are also known from diversity studies performed on isolates collected from soils of various geographic origin: the 'Neo' strains from Italian and South American soils (Neo 1, 2, 6, 9, 15) as described by Busti *et al.* [15], several isolates from Ellinbank, Australia, (Ellin 5034, 5116, 5119) as described by Joseph *et al*. [16], and a Korean isolate D8-90T (AM690741), all of which share at least 99.3% 16S rRNA gene sequence identity with strain ID 139908^T^. None of the samples sequenced in environmental genomic survey and screening programs surpassed 92% sequence similarity with strain ID 139908^T^, indicating a lack of close links of these phylotypes to the species *C. acidiphila* or the genus *Catenulispora*.

Figure 1 shows the phylogenetic neighborhood of *C. acidiphila* strain ID 139908^T^ in a 16S rRNA based tree. All three 16S rRNA gene copies in the genome of strain D 139908^T^ are identical, and also match the previously published 16S rRNA sequence generated from DSM 20547 (AJ865857).

**Figure 2:**
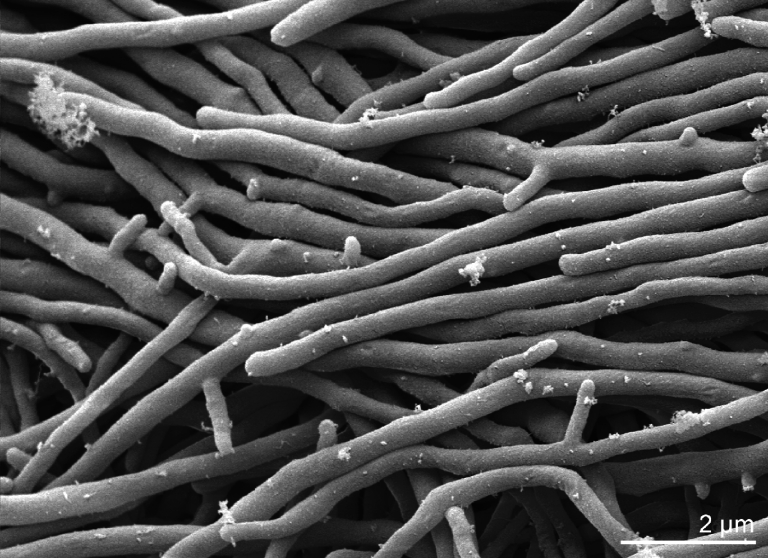
Scanning electron micrograph of *C. acidiphila* strain ID 139908^T^ (Manfred Rohde, Helmholtz Centre for Infection Research Braunschweig)

*C. acidiphila* strain ID 139908^T^ was described as a Gram-positive, acidophilic, non-acid fast, non-motile, essentially aerobic bacterium forming both vegetative and aerial mycelia [1] (Figure 2 and Table 1). Non-fragmentary vegetative mycelium and aerial hypha are straight to slightly flexuous and start to septate in chains of cylindrical arthrospores with a rugose surface when sporulation is induced [1]. Strain ID 139908^T^ grows on different agar media while producing brownish pigments and a whitish aerial mass which turned to yellow/green with the aging of bacteria [1]. The brownish pigments were not observed on tyrosine-supplemented Suter medium which indicated that they are not melanin-related [1]. The strain grows well in the presence of 3% (w/v) NaCl with a progressive reduction of pigmentation which started at 1% NaCl. Strain ID 139908^T^ grows better under aerobic conditions but is capable of reduced and non pigmented growth under microaerophilic and anaerobic conditions [1]. It is resistant to lysozyme (at least 100μg/ml) [1] which was not reported for any of the strains of the genus *Catenulispora*. Optimum temperature for growth was 22-28°C and the pH for growth ranges from 4.3 to 6.8 with an optimum pH level 6.0 but scant growth was reported up to pH 7.5 [1]. The organism is able to hydrolyze starch and casein, liquefy gelatin, and to utilize D-galactose, D-fructose, arabinose, xylose and gluconate but not glycerol, L-arabinose, D-mannitol, methyl-β-D-xylopyranoside, methyl-α-D-glucopyranoside, cellulose or sucrose [1].

### Chemotaxonomy

 Like the other *Catenulispora* strains [7,8], the murein of *C. acidiphila* strain ID 139908^T^ contains LL-diaminopimelic acid, glycine, glutamic acid and alanine [1] and can be assigned to type A3γ LL-Dpm–Gly. Whole-cell sugars contains large amounts of arabinose, together with xylose, ribose, rhamnose and glucose [1]. The predominant menaquinones in strain ID 139908^T^ contain nine isoprene units: MK-9(H_6_), -9(H_4_), and MK -9(H_8_) in a ratio of 4.5:2.8:1 [1], as also reported for other members of the genus [7,8]. As in *C. rubra* [7] and in *C. subtopica* and *C. yoronensis* [8], the major cellular fatty acids are iso- (i-) and anteiso- (ai-) branched chain saturated acids: i-C_16:0_ (47.1%) and ai-C_17:0_ (12.7%), with smaller amounts of i-C_17:0_ (5.7%), C_16:0_ (5.6%), i-C_17:1_ ω 9c (4.7%), i-C_15:0_ (4.3%), i-C_16:1_ (3.4%), C_16:1_ω7c (3.2%), ai-C_17:1_ ω 9c (2.8%), ai-C_15:0_ (2.3%) [1]. Phosphatidylglycerol, diphosphatidylglycerol, phosphatidyl-inositol, phosphatidylinositol mannosides were identified as the dominant polar lipids together with two unknown phospholipids [1].

## Genome sequencing and annotation

### Genome project history

This organism was selected for sequencing on the basis of its phylogenetic position, and is part of the *** G****enomic* *** E****ncyclopedia of* *** B****acteria and* *** A****rchaea * project. The genome project is deposited in the Genomes OnLine Database [6] and the complete genome sequence in GenBank. Sequencing, finishing and annotation was performed by the DOE Joint Genome Institute (JGI). A summary of the project information is shown in Table 2.

**Table 2:** Genome sequencing project information

**MIGS ID**	**Property**	**Term**
MIGS-31	Finishing quality	Finished
MIGS-28	Libraries used	Two Sanger libraries - 8 kb pMCL200 and fosmid pcc1Fos
MIGS-29	Sequencing platforms	ABI3730
MIGS-31.2	Sequencing coverage	10× Sanger
MIGS-30	Assemblers	Phred/Phrap/Consed
MIGS-32	Gene calling method	Prodigal, GenePrimp
	INSDC / Genbank ID	CP001700
	Genbank Date of Release	August 26, 2009
	GOLD ID	Gc01085
	NCBI project ID	21085
	Database: IMG-GEBA	2501533203
MIGS-13	Source material identifier	DSM 44928
	Project relevance	Tree of Life, GEBA

### Growth conditions and DNA isolation

*C. acidiphila* strain ID 139908^T^ (DSM 44928) was grown in DSMZ medium 65 (GYM Streptomycetes Medium) at 28°C. DNA was isolated from 0.5-1 g of cell paste using the JGI CTAB protocol with lysis modification ALM as described in Wu *et al*. [17].

### Genome sequencing and assembly

The genome was sequenced using the Sanger sequencing platform only. All general aspects of library construction and sequencing performed can be found at the JGI website. The Phred/Phrap/Consed software package was used for sequence assembly and quality assessment. After the shotgun stage, reads were assembled with parallel phrap (High Performance Soft ware, LLC). Possible mis-assemblies were corrected with Dupfinisher [18] or transposon bombing of bridging clones (Epicentre Biotechnologies, Madison, WI). Gaps between contigs were closed by editing in Consed, custom primer walking or PCR amplification (Roche Applied Science, Indianapolis, IN). A total of 2,556 finishing reactions were produced to close gaps and to raise the quality of the finished sequence. The completed genome sequences of *C. acidiphila* contains 126,099 Sanger reads, achieving an average of 10x sequence coverage per base with an error rate less than 1 in 100,000.

### Genome annotation

Genes were identified using Prodigal [19] as part of the Oak Ridge National Laboratory genome annotation pipeline, followed by a round of manual curation using the JGI GenePRIMP pipeline [20]. The predicted CDSs were translated and used to search the National Center for Biotechnology Information (NCBI) nonredundant database, UniProt, TIGRFam, Pfam, PRIAM, KEGG, COG, and InterPro databases. Additional gene prediction analysis and functional annotation was performed within the Integrated Microbial Genomes Expert Review (IMG-ER) platform [21].

## Genome properties

**Figure 3:**
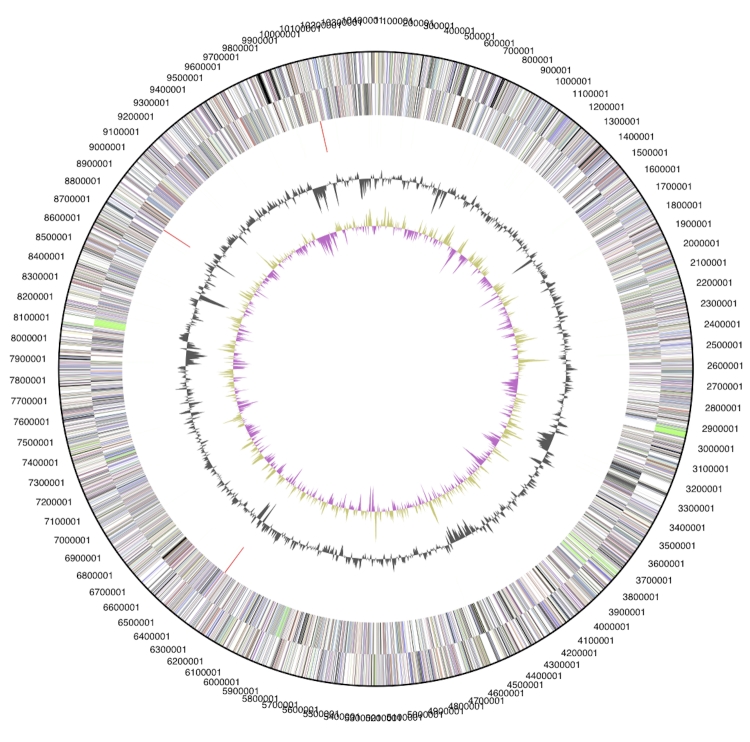
Graphical circular map of the genome. From outside to the center: Genes on forward strand (color by COG categories), Genes on reverse strand (color by COG categories), RNA genes (tRNAs green, rRNAs red, other RNAs black), GC content, GC skew.

The genome is 10,467,782 bp long and comprises one circular chromosome with a 69.8% GC content (Table. 3 and Figure 3). Of the 9,122 genes predicted, 9,056 were protein coding genes and 66 RNAs. In addition, 142 pseudogenes were also identified. Of the genes discovered, 68.2% were assigned with a putative function while the remaining genes were annotated as hypothetical proteins. The properties and the statistics of the genome are summarized in Table 3. The distribution of genes into COG functional categories is presented in Figure 3 and Table 4.

**Table 3:** Genome Statistics

**Attribute**	Value	% of Total
Genome size (bp)	10,467,782	100.00%
DNA Coding region (bp)	9,386,056	89.67%
DNA G+C content (bp)	7,303,066	69.77%
Number of replicons	1	
Extrachromosomal elements	0	
Total genes	9122	100.00%
RNA genes	66	0.76%
rRNA operons	3	
Protein-coding genes	9056	99.28%
Pseudo genes	142	1.56%
Genes with function prediction	6226	68.25%
Genes in paralog clusters	2379	26.08%
Genes assigned to COGs	5805	63.64%
Genes assigned Pfam domains	6202	67.99%
Genes with signal peptides	2279	24.98%
Genes with transmembrane helices	2231	24.46%
CRISPR repeats	4	

**Table 4:** Number of genes associated with the general COG functional categories

**Code**	**Value**	**%age**	**Description**
J	182	2.0	Translation, ribosomal structure and biogenesis
A	2	0.0	RNA processing and modification
K	607	6.7	Transcription
L	173	1.9	Replication, recombination and repair
B	2	0.0	Chromatin structure and dynamics
D	34	0.4	Cell cycle control, mitosis and meiosis
Y	0	0.0	Nuclear structure
V	96	1.1	Defense mechanisms
T	389	4.3	Signal transduction mechanisms
M	210	2.3	Cell wall/membrane biogenesis
N	45	0.5	Cell motility
Z	1	0.0	Cytoskeleton
W	0	0.0	Extracellular structures
U	46	0.5	Intracellular trafficking and secretion
O	149	1.6	Posttranslational modification, protein turnover, chaperones
C	306	3.4	Energy production and conversion
G	441	4.9	Carbohydrate transport and metabolism
E	425	4.7	Amino acid transport and metabolism
F	108	1.2	Nucleotide transport and metabolism
H	223	2.5	Coenzyme transport and metabolism
I	226	2.5	Lipid transport and metabolism
P	241	2.7	Inorganic ion transport and metabolism
Q	265	2.9	Secondary metabolites biosynthesis, transport and catabolism
R	670	7.4	General function prediction only
S	328	3.6	Function unknown
-	3251	35.9	Not in COGs
